# Vascular Protection of Poly(ADP-ribose) Polymerase Inhibitors in the Combination Therapy With Vascular Endothelial Growth Factor Signaling Pathway Inhibitors

**DOI:** 10.31083/RCM44942

**Published:** 2026-03-12

**Authors:** Jie Ma, Caie Li, Wenjuan Wang, Xin Fan, Taotao Wei, Xin Ma, Yingdong Wang, Chuyan Feng, Jing Yu

**Affiliations:** ^1^Department of Hypertension Center, Lanzhou University Second Hospital, 730030 Lanzhou, Gansu, China; ^2^Department of Cardiovascular Center, The First People’s Hospital of Huzhou City, 313000 Huzhou, Zhejiang, China

**Keywords:** PARPIs, vascular endothelial growth factor receptor inhibitors, PARP1, hypertension

## Abstract

The Poly(ADP-ribose) polymerase (PARP) family comprises seventeen members that catalyze poly- or mono- adenosine diphosphate (ADP)-ribosylation, a pivotal post-translational modification regulating a wide array of cellular processes, including deoxyribonucleic acid (DNA) repair, apoptosis, protein synthesis, cellular proliferation, and responses to oxidative stress. PARP inhibitors (PARPIs) exhibit selective cytotoxicity in cancers with breast cancer susceptibility gene (*BRCA*) mutations or defects in homologous recombination. Activation of PARP, indicated by increased poly(ADP-ribose) (PAR) accumulation, is implicated in various disease states such as ischemia-reperfusion injury, vascular disorders, and diabetic complications. Clinically, PARPIs, in combination with anti-angiogenic therapies, not only show efficacy as monotherapies in epithelial ovarian cancer but also mitigate hypertension induced by anti-angiogenic agents. This review consolidates recent advancements in understanding the dual therapeutic potential of PARP inhibition, encompassing both antineoplastic and cardioprotective effects.

## 1. Introduction

Hypertension represents a highly prevalent cardiovascular disorder and a major determinant of cardypertension is a prevalent cardiovascular disorder and a major contributor to cardiovascular risk. Emerging evidence has highlighted a significant association between malignancy and the development of hypertension [1, 2]. Cancer and hypertension not only share common risk factors but also exhibit overlapping pathophysiological mechanisms, leading to the emergence of “Onco-hypertension” as a distinct interdisciplinary field [3]. Although anti-neoplastic agents targeting the vascular endothelial growth factor (VEGF) pathway extend survival in cancer patients, their tendency to induce hypertension and subsequent cardiovascular complications can compromise both survival and quality of life, potentially negating the therapeutic benefits of anti-tumor treatments [2]. Therefore, understanding the mechanisms of anti-cancer therapy-induced hypertension and developing effective management strategies have become critical priorities in oncology cardiology.

Poly(ADP-ribose) polymerase (PARP) inhibitors (PARPIs), initially developed as PARP1/2-targeting anti-cancer agents, have shown substantial cardiovascular protective effects in numerous preclinical studies, positioning them as promising dual-purpose therapies for onco-hypertension management. This review focuses on the dual role of PARP1 in both oncological and cardiovascular contexts.

## 2. Poly(ADP-ribose) Polymerase (PARP) and PARP Inhibitors (PARPI)

Since its discovery over fifty years ago, the PARP family has been recognized as comprising seventeen distinct enzymes that mediate ADP-ribosylation, a crucial post-translational modification that regulates a wide range of cellular processes, including deoxyribonucleic acid (DNA) damage response, apoptosis, and adaptive stress signaling [4, 5]. Among these, PARP1, PARP2, and PARP3 are classified as DNA damage-sensing PARPs due to their essential roles in maintaining genomic stability [4, 6, 7]. Functionally, PARP1 and PARP2 catalyze the synthesis of extended, branched poly(ADP-ribose) chains (PARylation), while PARP3 primarily mediates mono(ADP-ribosyl)ation. Beyond their well-established involvement in DNA repair, several other PARP family members contribute to diverse cellular pathways, emphasizing their multifunctional roles in cellular physiology [8].

PARP1, the primary mediator of DNA damage response and repair within the PARP family, is ubiquitously expressed across eukaryotes. The human PARP1 protein consists of 1014 amino acids and has a molecular mass of 113 kDa. Its modular structure includes three zinc finger domains (ZnFI, ZnFII, ZnFIII), a BRCA1 C-terminal (BRCT) motif, a Trp-Gly-Arg (WGR) domain, and a C-terminal catalytic domain (CAT), which contains both an alpha-helical subdomain (HD) and an ADP-ribosyl transferase subdomain (ART) [9, 10]. These structural components perform specialized molecular functions that enable PARP1 to regulate critical cellular activities such as DNA repair, genomic integrity preservation, and apoptosis regulation.

PARPIs have received regulatory approval from agencies like the U.S. Food and Drug Administration (FDA) and the European Medicines Agency (EMA) for clinical use in several malignancies. Their current indications include ovarian, breast, pancreatic, and prostate cancers, where they exert therapeutic effects through synthetic lethality in tumors with breast cancer susceptibility gene (*BRCA*) mutations or homologous recombination deficiencies (HRDs) [11, 12, 13]. In addition to their established anti-cancer activity, emerging evidence suggests that PARPIs may offer therapeutic potential for non-oncological conditions associated with oxidative stress and vascular inflammation, particularly in cardiovascular and neurological diseases [14, 15].

## 3. Mechanistic Insights

### 3.1 PARPs Play an Important Role in Vascular Health

PARP enzymes play a significant role in the pathogenesis of cardiovascular disorders [16]. In conditions such as hypertension, hypertension-induced organ damage, atherosclerosis, acute myocardial infarction, and heart failure, disrupted energy metabolism within cardiomyocytes and endothelial cells constitutes a key mechanism underlying disease progression and tissue injury [17, 18, 19]. A recent meta-analysis assessed the cardioprotective potential of PARP inhibition in preclinical models, demonstrating that PARP inhibitors (PARPIs) improve various cardiac function indices, including cardiac output and stroke volume, while enhancing myocardial contractility [20].

PARP1 activation in vascular smooth muscle cells (VSMCs) has been implicated in the pathogenesis of vascular calcification [21, 22]. This pathological mineralization occurs in both the intimal and medial layers of arteries, each with distinct clinical implications. Intimal calcification is primarily associated with arterial obstruction and destabilization of atherosclerotic plaques, while medial calcification contributes to increased vascular stiffness, elevated systolic blood pressure, and accelerated pulse wave velocity—hemodynamic changes that promote diastolic dysfunction and heart failure [14, 23, 24]. Multiple studies confirm PARP activation at calcification sites in both human specimens and experimental models, where significant accumulation of poly(ADP-ribose) (PAR) colocalizes with DNA damage markers [25]. Under oxidative stress, excessive PARP1 activation depletes cellular nicotinamide adenine dinucleotide (NAD^+^) and adenosine triphosphate (ATP) reserves, promotes PAR polymer accumulation, and triggers mitochondrial-nuclear translocation of apoptosis-inducing factor (AIF) [26]. A key event in vascular calcification is the phenotypic transition of VSMCs from contractile to osteogenic lineages [23]. In ApoE^-⁣/-^ mice, PARP1 signaling modulates runt-related transcription factor 2 (Runx2) protein levels, a central regulator of osteochondrogenic transdifferentiation. Mechanistically, overexpression of PARP1 downregulates miRNA-204 expression *via* the IL-6/STAT3 axis, relieving Runx2 repression and enhancing its protein stability [14, 25]. Additionally, PARP1-mediated PARylation of DNA polymerase gamma (POLG) promotes iron-induced vascular calcification through activation of the Adora2a/Rap1 signaling pathway [27]. These processes position vascular calcification as a crucial component of atherosclerotic progression and a recognized contributor to plaque vulnerability.

Furthermore, PARP1 has been established as a significant factor in atherosclerosis pathogenesis [28]. Evidence suggests that vascular inflammation and endothelial dysfunction drive PARP1 overactivation in this context [29, 30, 31]. In a study using apolipoprotein E-deficient mice, a well-established model of atherosclerosis, Von Lukowicz *et al*. [15] demonstrated that pharmacological PARP inhibition or genetic PARP1 deletion reduced plaque formation. Their findings also revealed that PARP1 upregulates adhesion molecule expression and promotes features of plaque vulnerability [15].

PARP1 has also been implicated in the pathogenesis of other vascular diseases, such as pulmonary arterial hypertension. Meloche *et al*. [32] demonstrated that in the context of inflammation, DNA damage triggers excessive PARP1 activation, which not only facilitates DNA repair to support the unchecked proliferation of pulmonary artery smooth muscle cells but also modulates the miR-204/NFATc2/HIF-1α signaling axis, further promoting proliferative responses. These coordinated actions ultimately contribute to the development and progression of pulmonary hypertension [32].

**Fig. 1.**
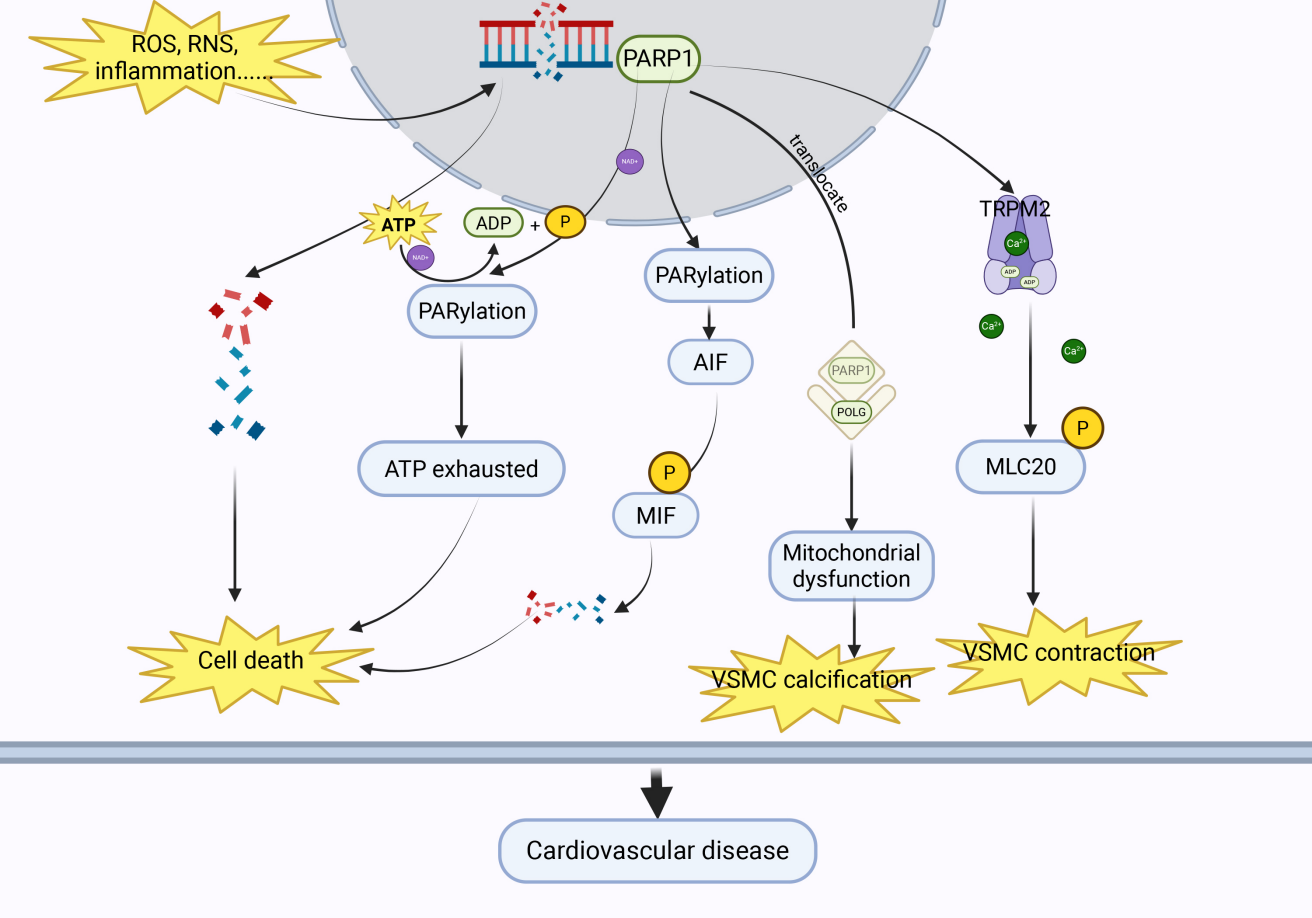
**Under conditions such as oxidative stress and inflammation, PARP1 can be activated, triggering cardiovascular diseases through multiple pathways**. PARP1, poly-ADP-ribose polymerase 1; ROS, reactive oxygen species; RNS, reactive nitrogen species; ATP, adenosine triphosphate; ADP, adenosine diphosphate; AIF, apoptosis-inducing factor; MIF, migratory inhibitory factor; TRPM2, transient receptor potential cation channel, subfamily M, member 2; VSMC, vascular smooth muscle cell; POLG, DNA polymerase gamma. Created with BioRender.com (https://www.biorender.com/).

Additionally, PARP1 is involved in oxidative stress-mediated vascular injury. In a study by Zhang *et al*. [33], which investigated angiotensin II-induced vascular damage, elevated oxidative stress was accompanied by increased expression of both Sirtuin 2 (SIRT2) and PARP1. Mechanistic analysis revealed that SIRT2 mediates the deacetylation of PARP1 and facilitates its ubiquitination through the recruitment of the E3 ubiquitin ligase WW Domain-Containing E3 Ubiquitin Protein Ligase 2 (WWP2), thereby regulating the degradation of PARP1 [33]. Fig. 1 illustrates the mechanism by which PARP1 mediates vascular injury.

### 3.2 PARPI Showed Vascular Protection in Combination With Vascular Endothelial Growth Factor Signaling Pathway Inhibitors 

#### 3.2.1 Hypertension Caused by Vascular Endothelial Growth Factor Signaling Pathway Inhibitors

Anti-angiogenic therapies primarily target the VEGF signaling pathway and include both small-molecule tyrosine kinase inhibitors (e.g., Sunitinib, Apatinib) and monoclonal antibodies (e.g., Bevacizumab) [2]. However, these VEGF signaling pathway inhibitors (VSPIs) are associated with significant cardiovascular toxicities, including hypertension and acute kidney injury. Hypertension is the most commonly observed adverse effect, with its severity sometimes requiring treatment interruption due to hypertensive emergencies [34, 35, 36]. The incidence of hypertension in patients treated with VSPIs, either alone or in combination, ranges from 20% to 90%, while 6% to 43% of patients experience severe hypertension [37, 38]. Interestingly, PARPIs have been shown to alleviate hypertension induced by VSPIs.

#### 3.2.2 Potential Protective Mechanisms of PARPI in VSPI-Induced Hypertension

Endothelial dysfunction is a key mechanism underlying VSPI-induced hypertension (VSPI-HTN). VSPIs impair endothelial integrity through several pathways, primarily by reducing the production of vasodilatory mediators such as endothelial nitric oxide synthase (eNOS). This disruption in endothelial homeostasis skews the balance between vasodilation and vasoconstriction, ultimately resulting in elevated blood pressure [2]. PARPIs counteract these effects by enhancing the tyrosine phosphorylation of vascular endothelial growth factor receptor 2 (VEGFR2) and activating the AKT serine/threonine kinase (AKT) signaling pathway, thereby promoting endothelial cell survival. Experimental evidence shows that both PJ34 hydrochloride (a PARP1/2 inhibitor) and PARP1 siRNA effectively attenuate reactive oxygen species (ROS)- and reactive nitrogen species (RNS)-induced nicotinamide adenine dinucleotide (NAD^+^) depletion, ATP loss, and subsequent endothelial cell death. Additionally, PARP inhibition enhances AKT phosphorylation, further boosting pro-survival signaling networks [39]. Further mechanistic studies indicate that under oxidative stress conditions, PARP1 activation promotes the expression of ataxia-telangiectasia mutated (ATM) protein *via* PARylation. PARP inhibition disrupts this modification, facilitating the formation of an ATM- NF-κB essential modulator (NEMO) complex that interacts with mechanistic target of rapamycin (mTOR) and AKT in the cytosol. This multiprotein complex promotes AKT phosphorylation, initiating cytoprotective signaling through AKT-mediated survival pathways [40]. Together, these findings suggest that PARPIs ameliorate VSPI-HTN through modulation of the PARP/AKT axis.

**Fig. 2.**
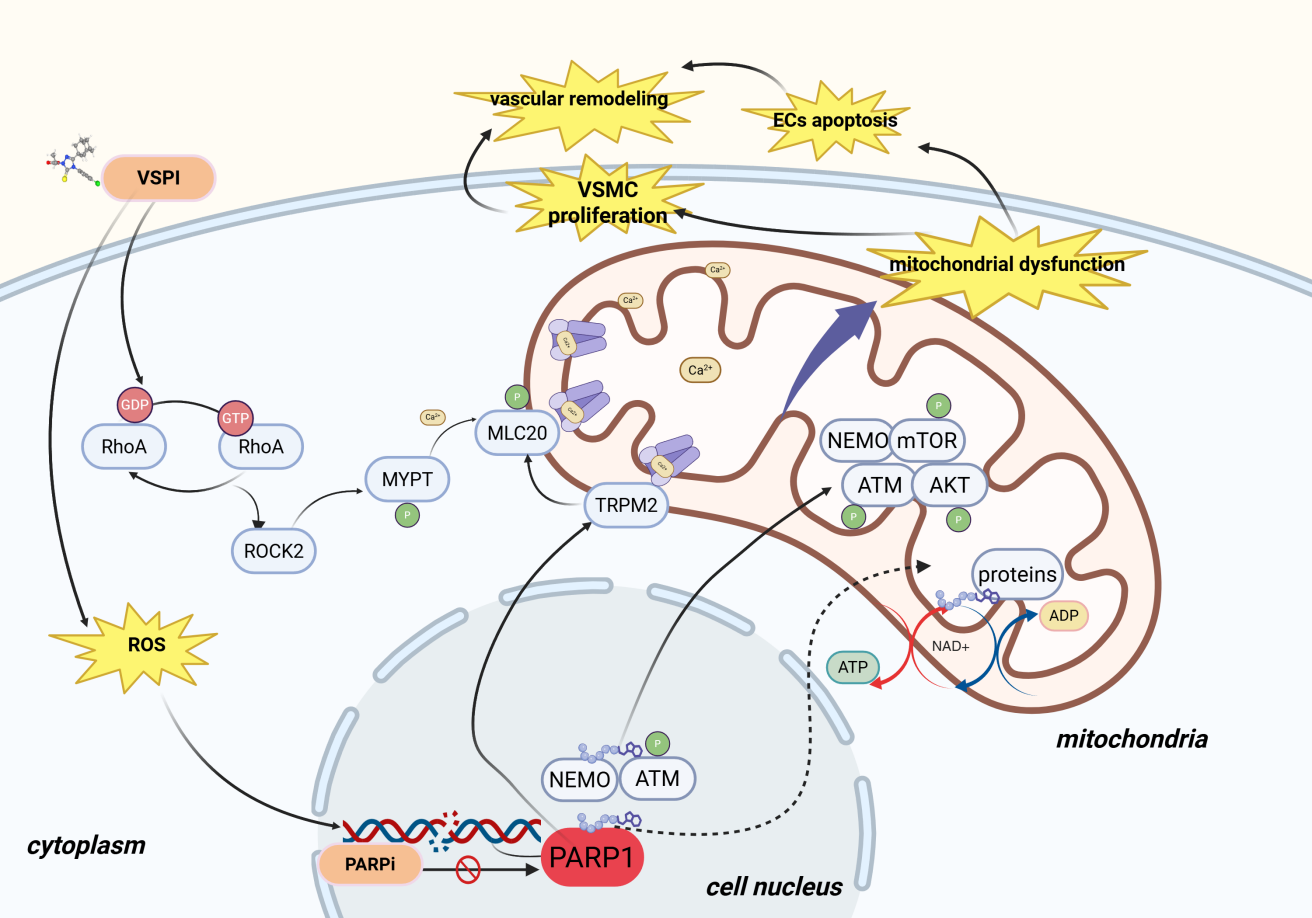
**PARP inhibitors alleviate vascular dysfunction induced by VSPIs through multiple pathways**. VSPI, vascular endothelial growth factor signaling pathway inhibitors; RhoA, ras homolog family member A; ROCK2, rho-associated coiled-coil containing protein kinase 2; MYPT, myosin phosphatase target subunit; GDP, guanosine diphosphate; GTP, guanosine triphosphate; NEMO, NF-kappa-B essential modulator; ATM, ataxia telangiectasia mutated; PARP1, poly(ADP-ribose) polymerase 1; TRPM2, transient receptor potential cation channel, subfamily M, member 2; AKT, AKT serine/threonine kinase; mTOR, mechanistic target of rapamycin; ATP, adenosine triphosphate; ADP, adenosine diphosphate; ROS, reactive oxygen species; NAD^+^, nicotinamide adenine dinucleotide; ECs, endothelial cells; PARP1, poly-ADP-ribose polymerase 1. Created with BioRender.com (https://www.biorender.com/).

Vascular remodeling is a fundamental pathological process in VSPI-HTN. Our previous studies demonstrated that Apatinib promotes abnormal VSMC proliferation, significantly increasing the media-to-lumen ratio and raising blood pressure [41, 42]. Neves *et al*. [43] found that the PARPI Olaparib improved blood vessel remodeling induced by Axitinib through the PARP/TRPM2 signaling pathway, which in turn mitigated the increase in blood pressure caused by Axitinib [43]. As summarized in Fig. 2, these findings collectively suggest that PARPIs mitigate VSPI-mediated vascular damage through multiple molecular pathways.

In the pathogenesis of hypertension, angiotensin II signaling through endothelial receptors enhances NADPH oxidase activity, leading to the generation of superoxide and peroxynitrite, which induce DNA strand breaks and activate PARP [44]. However, the mechanisms underlying VSPI-HTN differ from those of primary hypertension. Previous studies have identified that Rho/ROCK pathway activation contributes to Apatinib-induced hypertension and renal vascular remodeling by suppressing the renin-angiotensin system (RAS). ROCK inhibition alleviates hypertension by increasing angiotensin-converting enzyme 2 (ACE2) activity, promoting the production of the vasodilatory and anti-remodeling peptide Ang-(1-7), and inhibiting vascular and renal pathological remodeling [45]. However, other studies have suggested that VSPIs induce hypertension through mechanisms independent of RAS [34]. The role of RAS in VSPI-HTN warrants further investigation. In conclusion, the precise mechanisms by which PARPIs improve VSPI-induced vascular dysfunction and elevated blood pressure require clarification, representing a promising area of research in onco-cardiology.

#### 3.2.3 Niraparib Showed Vascular Damage due to Its Off-Target Effects

PARPIs, particularly Olaparib, have demonstrated protective effects in various contexts. However, Niraparib, another PARPI, has been observed to induce hypertension in clinical trials, primarily due to off-target effects [46, 47]. According to FDA notes on Niraparib, hypertension may arise because Niraparib binds to dopamine, norepinephrine, and serotonin transporters, inhibiting the intracellular uptake of dopamine and norepinephrine [48]. Both Niraparib and Rucaparib have also been linked to arrhythmogenic potential in clinical reports, a risk attributed to their inhibition of the Kv11.1 potassium channel (hERG), which delays cardiac repolarization and predisposes patients to QT interval prolongation, a known risk factor for torsades de pointes. Both Niraparib and Rucaparib share structural characteristics common to hERG inhibitors, being weakly basic compounds with molecular features that facilitate interaction with the channel. In contrast, Olaparib presents a more favorable cardiac safety profile, with no documented arrhythmogenic events in clinical data and a lack of structural determinants associated with hERG channel blockade [49].

### 3.3 PARP1 Is Involved in the Occurrence of Other Cardiovascular Diseases

PARP1 plays a significant role in the pathogenesis of ventricular hypertrophy [50, 51]. Chronic pharmacological inhibition of nuclear PARP activity reduces ADP-ribosylation of nuclear proteins, thereby attenuating the progression toward heart failure [52]. Additionally, PARPIs protect against hypertensive cardiomyopathy by preserving mitochondrial integrity and bioenergetics. Specifically, PARPIs prevent oxidative stress-induced mitochondrial fragmentation, promote fusion-favoring dynamics, enhance mitochondrial biogenesis, and maintain ultrastructural organization, all of which collectively sustain cellular energy production [17]. At the molecular level, Zhang *et al*. [53, 54] identified a regulatory mechanism in which BRG1/BRM-Associated Factor 155 (BAF155) facilitates maladaptive cardiac remodeling by suppressing (WWP2)-mediated PARP1 ubiquitination and subsequent proteasomal degradation, thereby stabilizing PARP1 and promoting its hypertrophic actions. Myb-like, SWIRM and MPN Domains 1 (MYSM1) mediates cardiac hypertrophy through PARP1-dependent cardiomyocyte parthanatos [55]. These findings highlight the critical role of PARP in ventricular hypertrophy induced by elevated blood pressure, particularly through disruption of mitochondrial structure, and suggest that PARPIs may mitigate this condition. Therefore, further exploration of the role of PARPIs in cardiovascular structural changes presents an intriguing avenue for future research.

## 4. Clinical Efficacy of PARPI

Ovarian cancer remains a life-threatening gynecological malignancy with significant global health consequences. Epidemiological data show an annual incidence of 313,959 cases for ovarian, fallopian tube, and peritoneal cancers combined, resulting in approximately 207,252 deaths. Projections indicate that by 2025, the United States will report 20,890 new cases of ovarian cancer, with a death toll of 12,730 [56]. The clinical course of this disease is often marked by recurrence following initial cytoreductive surgery and platinum-based chemotherapy, leading to disease progression and mortality—highlighting the urgent need for novel therapeutic strategies [57, 58]. Ovarian carcinogenesis is characterized by significant genomic instability, often manifesting as deficiencies in DNA damage repair mechanisms. Notably, defects in homologous recombination (HR) repair, frequently associated with mutations in breast cancer susceptibility genes *BRCA1* or *BRCA2*, create therapeutic vulnerabilities that can be targeted by specific agents [21].

PARPIs exploit these defects through mechanisms of synthetic lethality and PARP trapping to exert potent antitumor effects [59]. In the presence of BRCA defects, PARPIs impair the repair of single-strand breaks (SSBs) and double-strand breaks (DSBs), leading to cell death *via* synthetic lethality. PARPIs also induce cytotoxicity by trapping PARP1/PARP2 at DNA damage sites. PARP trapping refers to the ability of PARPIs to lock PARP1 onto DNA, disrupting the DNA repair process and causing the accumulation of toxic repair intermediates, ultimately resulting in cell death [59, 60]. Several PARPIs, including Olaparib, Niraparib, and Rucaparib, have received sequential regulatory approvals from both the FDA and EMA for clinical use in ovarian cancer [21].

## 5. Side Effects of PARPI

### 5.1 All Kinds of Side Effects of PARPI

Adverse events associated with the antitumor effects of PARPIs are typically mild to moderate (CTCAE grades 1 or 2) and can generally be managed without the need for dose reduction, treatment delay, or discontinuation. Meta-analyses indicate that the most common side effects of PARPIs include fatigue, gastrointestinal issues, and hematological toxicity [61].

### 5.2 Cardiovascular Side Effects 

A meta-analysis of 32 randomized controlled trials (RCTs) revealed that PARPI therapy was associated with major adverse cardiovascular events (MACEs) of 5.0% for any grade and 0.9% for high grade, compared to 3.6% and 0.9%, respectively, in control groups. For hypertension, PARPIs demonstrated an incidence of 17.5% for any grade and 6.0% for high grade, compared to 12.6% and 4.4% in controls. PARPI treatment significantly increased the risk of any-grade hypertension (random effects model: relative risk (RR) 1.53; *p* = 0.03), though this association was not statistically significant for high-grade hypertension (RR = 1.47; *p* = 0.09) [62]. Another meta-analysis involving 10,654 participants from 32 randomized trials further quantified the risk profile across the PARPI class. The pooled analysis showed incidence rates of 12% for all-grade hypertension (hazard ratio (HR) 1.22; 95% confidence interval (CI): 0.91–1.65; *p* = 0.19; I^2^ = 81%) and 4% for grade 3–4 hypertension (HR 1.24; 95% CI: 0.74–2.08; *p* = 0.42; I^2^ = 68%), neither of which reached statistical significance [63].

Actually, studies to date have confirmed that PARPI approved for clinical use rarely produce cardiovascular toxicity, and on the contrary, they showed cardiovascular protection, especially olaparib. However, hypertension associated with PARPIs is almost exclusively linked to Niraparib [2]. Clinical trials have demonstrated that Niraparib presents significant cardiovascular toxicity compared to other PARPIs. In the NOVA trial, 71 (19%) of 367 patients treated with Niraparib developed hypertension, compared to 8 of 179 (4.5%) in the placebo group. Additionally, 30 (8%) experienced grade 3 or 4 hypertension, and 38 (10%) reported palpitations of any grade [64]. A meta-analysis of 41 RCTs found that Niraparib significantly increased the risk of hypertension compared to placebo (RR 2.65 [95% CI: 1.28–5.49], *p* < 0.01) [65]. In contrast, other PARPIs have even been associated with hypotension. Furthermore, Niraparib has been linked to other adverse cardiovascular events, including arrhythmias. A summary of adverse cardiovascular event data from the FDA Adverse Event Reporting System (FAERS) covering the first quarter of 2015 to the second quarter of 2023 revealed that Niraparib reported a total of 5292 adverse events, 2308 of which were cardiovascular in nature, including hypertension, increased heart rate, arrhythmia, and thrombosis [66, 67].

Overall, while different types of PARPIs exhibit varying effects on cardiovascular protection, with the exception of Niraparib, the clinical use of PARPIs does not typically result in severe adverse cardiovascular events. Conversely, PARP1 activation has been associated with myocardial dysfunction, hypertension, and angiotensin-II activation, suggesting that PARPIs may have cardioprotective effects. Table 1 summarizes the incidence of cardiovascular toxicity associated with PARPIs and their corresponding drugs [62].

**Table 1.** **The probability of adverse cardiovascular events in PARPI and related drugs**.

Cardiovascular side effects	Incidence		Related drugs
	Any grade	Grade ≥3	
MACE	5.0%	0.9%	Olaparib, Niraparib, Rucaparib and Talazoparib
Hypertension	17.5%	6.0%	Niraparib
Thromboembolic events	4.1%	2.0%	Niraparib

Notes: MACE, major adverse cardiovascular events.

### 5.3 Risk Stratification and Patient Selection

Hypertension is the most common treatment-emergent adverse event associated with VSPIs [2, 68, 69]. Some studies suggest that hypertension induced during anti-tumor therapy targeting VEGF may serve as a biomarker for longer survival [70, 71]. However, no studies have yet explored whether hypertension caused by the PARPI Niraparib is associated with improved survival outcomes. It is generally believed that the increase in blood pressure resulting from Niraparib’s off-target effects may indicate a poor therapeutic response. Therefore, careful assessment of blood pressure should be performed prior to initiating Niraparib treatment, with decisions made based on the patient’s blood pressure condition.

## 6. Combining PARPI With VSPIs in Ovarian Cancer Treatment 

Despite the efficacy of PARPIs, resistance remains a significant challenge, particularly in patients with BRCA1/2 mutations, with over 40% of these patients showing suboptimal responses [72]. The development of resistance to PARPIs is primarily attributed to the following mechanisms: (i) restoration of HR, (ii) alterations in PARP1 activity and PAR levels, (iii) reduced cellular availability of PARPIs, and (iv) restoration of replication fork protection [73, 74]. In response to this challenge, combination anti-tumor regimens are being explored, and several randomized clinical trials are underway to assess the efficacy and safety of these therapies. To overcome resistance, combination therapies involving PARPIs with other agents, such as immunosuppressants, anti-angiogenic drugs, and chemotherapeutics, are under development. Among these, PARPI and anti-angiogenic drug combinations have shown promising anti-tumor effects while also mitigating the cardiovascular side effects typically associated with anti-angiogenic therapies [75].

The strategic combination of PARPIs with anti-angiogenic drugs has shown enhanced therapeutic efficacy in ovarian cancer [76, 77]. Recently, the FDA and EMA approved the combination of Olaparib and Bevacizumab as maintenance therapy for adults with advanced high-grade epithelial ovarian cancer (FIGO stages III–IV) exhibiting HRD positivity, who have achieved a complete or partial response following first-line platinum-based chemotherapy. This regulatory decision was supported by the PAOLA-1 trial [78, 79]. The PAOLA-1 trial, a phase III randomized controlled study, compared patients with high-grade serous and endometrial ovarian cancer who had a partial or complete response after platinum-taxane chemotherapy plus Bevacizumab, with those receiving either Bevacizumab alone or Bevacizumab combined with Olaparib for 24 months. The results showed a median progression-free survival (PFS) of 22.1 months in the Olaparib plus Bevacizumab group, compared to 16.6 months in the placebo plus Bevacizumab group. Surprisingly, the combination therapy group had a lower incidence of hypertension (18.8%) than the placebo plus Bevacizumab group (37.5%). This finding highlights the benefits of the combination treatment. Additional evidence from the OCTOVA trial further supports this approach, showing superior efficacy of the Olaparib-Cediranib combination over Olaparib monotherapy in platinum-resistant ovarian cancer. The combination therapy achieved a median PFS of 5.4 months, compared to 3.7 months with Olaparib alone, with benefits observed regardless of BRCA status, prior PARPI exposure, or previous anti-angiogenic therapy (HR = 0.73; 60% CI: 0.59–0.89; *p* = 0.1). Cediranib-associated hypertension of grades 1–2 occurred at expected frequencies in the combination arm but not in the control group [80]. However, this trial did not include a Cediranib-only group to confirm the efficacy and risk of hypertension associated with Cediranib alone. The NRG-GY005 trial assessed the efficacy of Cediranib, Olaparib, and their combination compared to standard-of-care chemotherapy in platinum-resistant or platinum-refractory epithelial ovarian cancer. Recent results from this trial indicated hypertension incidences of 65% and 75.3% in the Cediranib/Olaparib and Cediranib-only arms, respectively [81].

A meta-analysis of seven RCTs demonstrated that PARPI combination regimens significantly prolong PFS [82]. In a complementary pharmacovigilance study utilizing the FAERS database, Han *et al*. [83] showed that concurrent administration of PARPIs with chemotherapy/Bevacizumab regimens was associated with a substantially reduced risk of cardiac adverse events (reporting odds ratio (ROR) = 0.352; 95% CI: 0.194–0.637).

Notably, the combination of the next-generation PARPI Fluzoparib with the highly selective VEGFR2-targeted tyrosine kinase inhibitor Apatinib has demonstrated promising anti-tumor effects in animal models with tolerable side effects [84]. Clinical trials have also shown positive results with the Fluzoparib-Apatinib combination [85]. However, since the trial was prospective and not designed for direct comparison with control groups receiving either Fluzoparib or Apatinib alone, it is not possible to definitively assess the differences between the groups or the impact of Fluzoparib on vascular dysfunction caused by Apatinib.

Given the preliminary success of these combination therapies, further comprehensive trials are expected. These studies will aim to evaluate the superiority of these combinations over single-agent therapies and provide deeper insights into the mechanisms underlying their improved efficacy and safety profiles in treating ovarian cancer. Table 2 (Ref. [78, 86, 87, 88, 89, 90, 91]) summarizes ongoing clinical trials investigating combination therapy.

**Table 2.** **Prospective study of PARPI combination therapy**.

Study name	Study phase	Patient population	Study design
PAOLA-1 [78] (NCT02477644, 2015-06-18)	III	Newly diagnosed advanced high-grade serous or endometrioid ovarian cancer, primary peritoneal cancer, or fallopian-tube cancer.	Bevacizumab vs. Olaparib plus Bevacizumab
AVANOVA-2 [86] (NCT02354131, 2015-01-25)	II	Platinum-sensitive recurrent ovarian cancer.	Niraparib vs. Niraparib plus Bevacizumab
NCT01116648, 2010-04-29 [87]	II	Platinum sensitive ovarian cancer of high-grade serous or endometrioid histology or had a deleterious germline BRCA1/2 mutation.	Olaparib vs. Olaparib plus Cediranib
ICON9 [88] (NCT03278717, 2017-07-31)	III	High grade serous or endometrioid carcinoma of the ovary, fallopian tube or peritoneum.	Olaparib vs. Olaparib plus Cediranib
NRG‐GY012 [89] (NCT03660826, 2018-09-06)	II	Eligible patients were aged 18 years or older, had histologically confirmed recurrent or persistent endometrial cancer.	Olaparib vs. Cediranib vs. Cediranib plus Olaparib
OCTOVA [90] (NCT03117933, 2017-03-21)	II	Ovarian, fallopian tube, primary peritoneal cancer that had relapsed within 12 months of previous platinum-based therapy.	Paclitaxel vs. Olaparib vs. Olaparib plus Cediranib
AGO-OVAR28/ENGOT-ov57 [91] (NCT05009082, 2021-08-02)	III	Newly diagnosed, advanced high-grade epithelial ovarian cancer, primary peritoneal cancer, or fallopian tube cancer FIGO III/IV.	Niraparib vs. Niraparib plus Bevacizumab

Notes: The Table 2 summarizes the randomized controlled studies on the combined treatment of PARPI and VSPIs for ovarian cancer. VSPIs, vascular endothelial growth factor signaling pathway inhibitors.

## 7. Conclusion

PARPIs have emerged as dual-function agents with significant therapeutic potential in both oncology and cardiovascular medicine. Their clinical utility extends beyond single-agent applications, especially in combination regimens with anti-angiogenic therapies. These combinations not only enhance antitumor efficacy but also mitigate the cardiovascular side effects, particularly hypertension, commonly associated with anti-angiogenic monotherapy. However, the full therapeutic potential of PARP inhibition, both as monotherapy and in combination therapies, remains to be fully elucidated. Continued preclinical and clinical investigations are essential to comprehensively characterize the antitumor efficacy and cardioprotective properties of these compounds. Moreover, rigorously controlled clinical trials with long-term follow-up are necessary to confirm the sustained efficacy and safety of these combinations, ultimately establishing their superior clinical value compared to conventional therapeutic approaches.
